# A Systematic Review of Technology-Based Dietary Intake Assessment Validation Studies That Include Carotenoid Biomarkers

**DOI:** 10.3390/nu9020140

**Published:** 2017-02-14

**Authors:** Tracy L. Burrows, Megan E. Rollo, Rebecca Williams, Lisa G. Wood, Manohar L. Garg, Megan Jensen, Clare E. Collins

**Affiliations:** 1 Nutrition and Dietetics, School of Health Sciences, Faculty of Health and Medicine, University of Newcastle, Newcastle NSW 2308, Australia; Megan.rollo@newcastle.edu.au (M.E.R.); Rebecca.Williams@newcastle.edu.au (R.W.); Megan.jensen@newcastle.edu.au (M.J.); clare.collins@newcastle.edu.au (C.E.C.); 2 Priority Research Centre in Physical Activity and Nutrition, University of Newcastle, Newcastle NSW 2308, Australia; 3 School of Biomedical Sciences and Pharmacy, University of Newcastle, Newcastle NSW 2308, Australia; Lisa.wood@newcastle.edu.au (L.G.W.); Manohar.garg@newcastle.edu.au (M.L.G.)

**Keywords:** carotenoids, fruit vegetables, validation, biomarker

## Abstract

Technological advances have allowed for the evolution of traditional dietary assessment methods. The aim of this review is to evaluate the accuracy of technology-based dietary assessment methods to determine carotenoid and/or fruit and vegetable intake when compared with carotenoid biomarkers. An online search strategy was undertaken to identify studies published in the English language up to July 2016. Inclusion criteria were adults ≥18 years, a measure of dietary intake that used information and communication technologies that specified fruit and/or vegetable intake or dietary carotenoid, a biomarker of carotenoid status and the association between the two. Sixteen articles from 13 studies were included with the majority cross-sectional in design (*n* = 9). Some studies used multiple dietary assessment methods with the most common: food records (*n* = 7), 24-h diet recalls (*n* = 5), food frequency questionnaires (*n* = 3) and diet quality assessed by dietary screener (*n* = 1). Two studies were directly web based, with four studies using technology that could be completed offline and data later transferred. Two studies utilised technology in the collection of dietary data, while the majority (*n* = 11) automated the collection in combination with nutrient analysis of the dietary data. Four studies provided correlation values between dietary carotenoids with biomarkers, ranging from *r* = 0.13 to 0.62 with the remaining studies comparing a measure of fruit and vegetable intake with biomarkers (*r* = 0.09 to 0.25). This review provides an overview of technology-based dietary assessment methods that have been used in validation studies with objectively measured carotenoids. Findings were positive with these dietary assessment measures showing mostly moderate associations with carotenoid biomarkers.

## 1. Introduction

Technological advances in methods of collecting dietary intake data have been achieved in recent years with increased use and access of the internet and smartphones. These advances have allowed for an expansion and adaptation of traditional methods, allowing the collection of detailed dietary intake with lower costs and burden for researchers, clinicians and patients/participants by allowing more timely approaches to data analysis [1]. There has been an expansion into image-based methods using mobile devices and development of standardised images to assist in the estimation of portion sizes [2]. These advances in technology have an increasing tendency to allow for self-administered methods rather than interviewer administered or paper-based surveys [3]. With the rapid evolution of technology-based methods there is a need to ensure that these methods are both valid and reliable.

One important aspect of dietary intake is fruits and vegetables. Regular consumption of fruits and vegetables is associated with reduced risk of chronic disease such as specific cancers including breast, oesophageal and lung [4,5,6,7,8], reduced risk of coronary heart disease [9,10], stroke [11,12] and type 2 diabetes mellitus [13,14] and decreased risk of asthma incidence and exacerbation in adults and children [15,16]. Plant components such as fiber, phytochemicals and a range of vitamins and minerals, also contribute to these protective effects [17]. Carotenoids are powerful antioxidants and are obtained primarily from fruit and vegetables. Various carotenoids, including lycopene and β-carotene, have been heavily studied due to their documented associations with decreased risk of disease [18]. Carotenoids are obtained solely from the diet and can also provide useful biomarkers that can be objectively measured in plasma and used to validate dietary assessment tools [19,20].

Our previous review of traditional paper-based dietary assessment methods identified 142 studies demonstrated the popularity of plasma carotenoids as a dietary validation measure [18]. This review summarised the dietary intakes and plasma concentrations and their expected associations by dietary assessment method and provided a benchmark for dietary studies. It was highlighted that the most commonly assessed carotenoids from the diet and biochemically were β-carotene found in high concentrations. The associations between dietary measures and plasma concentrations were strongest for cryptoxanthin (*r* = 0.38, *n* = 35 studies) and lowest for α-carotene (*r* = 0.27, *n* = 73 studies). Food records had a tendency to have stronger correlations with plasma concentrations than other types of dietary assessment methods. To date, no reviews have synthesized information specifically on technology-based assessments.

The aim of this review was to evaluate the prominent characteristics of studies that compared carotenoid intake assessed by a technology-based dietary assessment method when compared with objective biomarkers of carotenoids. 

## 2. Materials and Methods

An online search strategy was undertaken to identify studies published in the English language up from 1975 to July 2016. The review methodology was registered with PROSPERO (ID number CRD42016047276).

As the initial step, six online databases were searched: CINAHL, Embase, Cochrane, MEDLINE, ProQuest, PubMed and Excerpta Medica. Key words used individually and in combination were dietary assessment OR food frequency questionnaire OR diet/dietary recall OR diet record OR weighed food record OR validity/validation AND carotene OR carotenoids OR fruit OR vegetable. Electronic searches were supplemented by manual cross-checking of the reference lists of relevant publications. All study designs were included with limits placed on searches for adults and English language.

**Figure 1:**
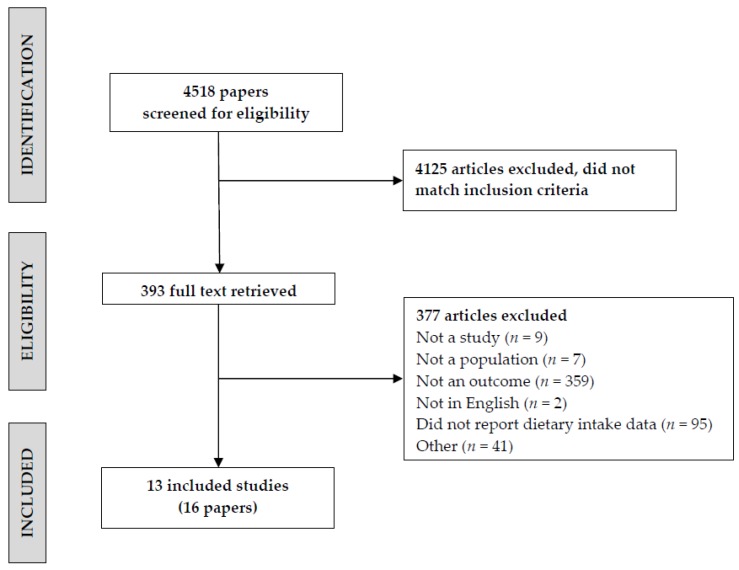
Flow diagram of article identification retrieval and inclusion for the systematic review.

After the removal of duplicates, stage 2 involved the assessment of titles and abstracts of identified studies by two independent reviewers with discrepancies decided by consensus using a third reviewer. A priori inclusion/exclusion criteria were applied to determine the eligibility of each publication for inclusion in the review, as per the following inclusion criteria: adult populations (≥18 years or “adults” depending on the database searched), a measure of dietary intake that specified fruit and/or vegetable, a measure of plasma or skin carotenoids as a biomarker of carotenoid intake, reported the association between diet and biomarker assessments. For the purpose of this review, dietary assessment methods which used information and communication technologies such as interactive programs based on the Internet or a computer [3] primarily to facilitate the collection of dietary intake data were included. This review focuses on carotenoids, individually or in combination, including α- and β-carotene, cryptoxanthin, lycopene, zeaxanthin, and lutein. Papers that met the inclusion criteria, or where eligibility was unclear, were retrieved. Studies were then evaluated for inclusion by two independent reviewers with discrepancies discussed with a third person. Excluded articles were classified in a systematized way and are summarized in Figure 1. i.e., “not an outcome” refers to a study not reporting information required for the review such as no correlation values.

Risk of bias was assessed using a standardized tool from the American Academy of Nutrition and Dietetics [21]. Ten quality criteria were rated as being absent, present or unclear in each study. This included the assessment of population bias, study blinding, a description of the intervention and assessment tool, statistical methods, and study funding. An overall quality rating was assigned, with each study being rated as positive, neutral or negative. No studies were excluded based on quality ratings.

Data were extracted using standardized tables developed for this review and included study design, population demographics, dietary assessment method, technology components (participant training, device used, self or interviewer administered, portion size tools, whether the method was collection only of diet or analysis only or a combination), carotenoids assessed and study outcomes. In cases of uncertainty regarding quality assessment, or data extraction, a third independent reviewer was consulted until consensus was reached. For studies that cited additional references with more details of the technology-based dietary assessment method, these additional references were retrieved.

## 3. Results

The search strategy identified 4518 articles, as summarised in Figure 1. Following elimination of duplicates, initial assessment of titles and abstracts, and evaluation of retrieved studies against the inclusion criteria, 16 articles from 13 studies were identified for critical appraisal and included in the review. The major reason for study exclusion was the dietary assessment method not being a technology-based method or not reporting associations between the outcome variables of diet and objectively measured carotenoids.

The majority of studies were conducted in the USA (*n* = 6 studies), France (*n* = 4), with one study each from the Netherlands, UK and Australia (Table 1). Nine studies were cross-sectional, three were cohort studies and one controlled trial. A total of 62,936 participants were included across the studies (mean 4841, range 91–17,688) with females only in three studies [22,23,24]. Five studies reported having recruited a diverse sample of participants from a range of ethnicities including African American, Hispanics and populations identifying as indigenous [24,25,26,27,28]. All studies except one [22,29] were published since the year 2000.

The quality assessment appraisals of included studies deemed that seven studies had a positive rating with six rated as having a neutral overall study quality (Supplementary Materials Table S1). As noted, many of the study designs were cross sectional so several of the quality criteria including ‘were study groups comparable’ were not applicable. Those studies which were rated as neutral did not describe details of participants who may have withdrawn from the study and lacked adequate descriptions of study methods.

### 3.1. Dietary Assessment Methods

In descending order, the most common technology-based dietary assessment methods used were: food records (*n* = 7), food recalls (*n* = 5 studies), food frequency questionnaires (*n* = 3), and diet quality/screener was assessed in one study using a pre-defined diet quality score [26]. The description of the collection of the dietary intake data using the record methods did not explicitly state if the collections occurred prospective or retrospective. Two studies utilised and employed two dietary methods [25,30] and one study employed three separate methods [22,29]. Only *n* = 3 studies assessed dietary intake data with a reporting a reporting longer than 24 h. Six studies specifically mentioned the assessment of supplements, one study reported intakes separately and not including supplements, while for the majority of studies (*n* = 6) it was unclear whether supplements were included or not. The nutrient database to evaluate dietary intakes was described in seven studies, with four studies not reporting any details and a further three studies reporting generic information such as “food tables”. Two studies used the USDA and one study each used AusNut, Minnesota, Ciqual and NutriNet.

Descriptive details of the technology-based dietary methods are detailed in Table 2. Training was provided to participants in three studies and in one study training was provided to interviewers. One study detailed that the inclusion criteria for the study was for participants to have basic computer knowledge [31]. Six methods were self-administered, three studies were interviewer administered, one study was a combination with some recalls collected under supervision with guidance and some self-administered [25], and in the remaining studies it was unclear as to which method was used. The specific technology device used was measured in four studies [29,32,33,34] with three of these using a console with Minitel and one the Portable Electronic Tape Recorded Automatic (PETRA) scales, which recorded the weight and a verbal description of the foods. Two studies were directly web based [25,31] while four studies stated that they could be completed offline and data later transferred [29,32,33,34]. Eight studies specifically reported that portion size was estimated using household measures or picture books [25,26,30,31,32,33,34,35]. Two studies utilised technology in the collection of dietary data, while the majority (*n* = 11) automated the collection in combination with nutrient analysis of the dietary data. The studies which automated collection and analysis of dietary data were all published from 2002 onwards, while only one study which used technology in the collection of dietary information and not analysis was published in 1995.

### 3.2. Dietary Carotenoids

Five studies reported dietary intakes as food groups, including fruit and vegetables [26,30,31,35], juices [32], or salad and vegetable consumption [28], however, these were all assessed and reported differently (i.e., grams per day, servings, amount of foods) preventing results to be pooled in a meta-analysis (Table 3). One study [24] reported the relative contribution of fruits and vegetables to carotenoid intakes, with the remaining studies reporting on intakes of individual dietary carotenoids. Of those reporting dietary carotenoids, two studies reported on five dietary carotenoids (α-carotene, β-carotene, lutein/zeaxanthin, lycopene and cryptoxanthin [25,27], three studies reported on β-carotene only [31,33,34] and one study reported the total amount of dietary carotene which was not specified further [29].

### 3.3. Carotenoid Biomarkers

Blood samples were collected from participants in a fasting state in nine studies, with three studies in a non-fasted state, however one of these was skin carotenoids which does not require fasting. For studies which reported plasma concentrations, high performance liquid chromatography (HPLC), which is considered the gold standard analytical technique for analysis of carotenoids, was used to assess plasma carotenoids in nine studies, absorptiometrics was used in one study, spectrophotometry in one, and the method was not reported in three studies.

Five carotenoids were assessed in six studies (α-carotene, β-carotene, lutein/zeaxanthin, lycopene and cryptoxanthin) [24,25,26,27,29,35], three carotenoids (α-, β-carotene and lycopene) were reported in one study [28] and β-carotene only assessed in four studies [31,32,33,34] (Table 3). One study also reported on total plasma carotenoids [30]. A study by Pezdirc et al. [23] reported on skin yellowness levels measured using reflectance spectroscopy, these were assessed across a range of sites including those which were sun exposed (i.e., shoulder) and non-exposed sites (i.e., sole of foot).

### 3.4. Correlations

Technology-based methods were compared to more traditional methods of dietary assessment in three studies [25,29,30]. The results reported by Arab et al. [25] demonstrated that a technology-based 24-h recall was more strongly correlated with plasma β-carotene, lutein + zeaxanthin, lycopene than a traditional diet history questionnaire in white but not African Americans. In another study by Bingham et al. [29] the technology-based PETRA weighed food record showed the strongest correlation (*r* = 0.45) compared to other methods which included a 7-day checklist, structured and unstructured 24-h recalls where correlations were *r* = 0.25, 0.06, and −0.01; respectively. In van Lee et al. [30], the technology-based 24-h recall had stronger correlations than the comparative method of an food frequency questionnaire (FFQ) for vegetables (*r* = 0.25 vs. 0.17) but not fruit (*r* = 0.09 vs. 0.25).

The four studies [22,25,27,34] that compared single dietary carotenoids with the corresponding plasma carotenoid biomarker at one time point demonstrated correlations for β-carotene ranging from 0.25–0.48 (*n* = 3 studies), α-carotene 0.21–0.62 (*n* = 4 studies), lycopene 0.13–0.33 (*n* = 3 studies), cryptoxanthin 0.37–0.51 (*n* = 2 studies) and lutein + zeaxanthin 0.35–0.45 (*n* = 2 studies). Those studies which reported fruit and vegetable intake and determined relationships with carotenoids tended to report on total carotenoids or carotenes so it cannot be ascertained which individual carotenoid biomarker was a predictor of fruit and vegetable intake. Studies that were in females only tended to produce similar correlation values with those in mixed gendered studies.

## 4. Discussion

This review evaluated the prominent characteristics of studies that compared carotenoid intake assessed by technology-based dietary assessment methods and carotenoid status from biomarkers. A total of 13 unique studies from 16 published papers were reviewed, each of which included a technology-based assessment of dietary intake and reported dietary intakes of fruits and vegetables, or carotenoids, and then compared these with a biomarker of carotenoid intake. 

The majority (>90%) of studies were published after 2002, indicating a growth in the use of technology for the assessment of dietary intake. This parallels the changes seen in society generally, with regard to access to and use of technology [43]. The internet has allowed enhancements to traditional approaches, such as shifts in the reliance on interviewer-administration of recalls to self-administration. This trend was evident in this review, with only three interviewer administered studies identified. Further, since the introduction of smartphones in the early 2000s, the development and use of technology-based applications has increased dramatically [2,44]. The original descriptions of dietary assessment methods, as summarised by Bingham (1987) [45], were predominantly paper-based, in-person and manual approaches to the collection and coding of intake data. Advances have seen the processing of collected dietary data via food composition software as standard in the analysis on nutrient intakes [46], attention has now shifted to improving efficiencies related to data collection. The majority of studies in the current review automated both the collection and analysis of dietary data through the use of various technologies. Overall, the included studies had relatively high participant numbers (mean 4841) when compared to other dietary validation studies (i.e., doubly labelled water were used [47]) where commonly fewer individuals (<20 per study) are used due to cost, technical skill and burden when technology was not employed in dietary assessment [18]. This may be attributed to the fact that when the collection of dietary intake data is facilitated by technology-based methods, it allows for substantial savings in time, greater scope in the size of the target population, in addition to reducing both participant and researcher burden. For example, the advent of web-based 24-h recall systems means that it is possible to collect 24-h recalls in large-scale undertakings, such as the ASA24, which previously would not have been possible [48].

The majority of studies used food records with a reporting period of 24 h being the most common, with few studies using methods such as FFQs. It is not clear why more methods that measure usual/habitual dietary intake rather than a short term intake were not used. Factors such as the high variation in the number of food items included in the food lists used in FFQs, in particular the increased number of fruit and vegetable items tend to be more strongly related to carotenoids and also the variability in the length of the reporting period may be attributed. FFQs also have to contain all possible or likely fruit and vegetable options whereas when diet is assessed by 24-h recall most people will have consumed a limited range of fruit and vegetables. In addition, FFQs are often generated for a particular group or population, therefore they cannot be as easily adapted to other settings such as a 24-h recall methodology. The majority of studies in this review compared plasma and dietary carotenoids directly. Four studies in this review compared plasma carotenoids with intakes of fruit, vegetables and/or juices with no study comparing a biomarker to specific types of fruits and vegetables which has been previously undertaken in children [20].

The development of technology-based, research tools for the assessment of intake, such as the web-based, automated, self-administer 24-h recall i.e., the ASA-24 [49] developed by the National Cancer Institute reflects the need, availability and popularity of smartphone applications and the popularity of wearable devices for self-monitoring intake [50]. In order to be confident in the data collected and inferences made by newer measures, one must ensure that any methods to be used for research purposes are valid and reliable [51].

Due to the accessibility of mobile devices and the high rates of use in both developed and developing countries [52], the use of technology-based methods has the potential to reach large populations and reduce language barriers through use of images rather than verbal descriptions. Technology allows for greater scale and efficiencies for researchers and government and non-government organisations relying on regular dietary intake data for surveillance and monitoring. It is important to note that although the conversion of paper-based methods into web-based methods may have benefits, including faster completion, greater reach, and the ability to maximise the collection of complete data, the methods do not address the limitations in terms of misreporting. Thus, there is a need for continued development of methods, as well as to continue to evolve statistical methods to mitigate error. Training research, clinical staff as well as patients on the use of the technology and the method is still highly warranted and will improve results and compliance to the dietary method. Examples of training might include taking images correctly and consistently to aid comparisons in addition to describing foods and remembering to record using the specified device. 

Food records were the most common type of dietary assessment method used across the studies included in this review. However for some studies, the description of the collection of intake data using the record methods does not explicitly state if collection occurred in as they were consumed or in real-time [53]. Initiatives such as the STROBE-nut which is a set of standardised guidelines for Strengthening the Reporting of Observational Studies in Nutritional Epidemiology) [54] may assist in improving the reporting of the methods used for the assessment dietary intake. In a previous review, FFQs were found to be the most common type of dietary assessment method used to comparatively validate with carotenoids FFQs were used in 103 of the 142 included studies [18]. FFQs were only used in three studies in the current review and, overall correlations were considered small to moderate. While much less studies were included in this review (*n* =16 studies), the correlations in this review are similar to that previously published on traditional paper-based dietary assessment methods (*n* = 142 studies). Specifically, in the previous review, the weighted mean correlation synthesised by meta-analysis for α-carotene was 0.34 (*n* = 41 studies) while the correlations in this review ranged from 0.21 to 0.62. Similarly, previously for β- carotene, the correlation *r* = 0.27 (*n* = 73 studies while in this review ranged 0.25 to 0.48; cryptoxanthin *r* = 0.38 (*n* = 35 studies) vs. in this review 0.37–0.51; lutein/zeaxanthin *r* = 0.29 (*n* = 28 studies) vs. in this review 0.35–0.45; lycopene *r* = 0.29 (*n* = 42) vs. in this review 0.13–0.33. The results from this review are promising and suggest that the collection of dietary data using technology provides similar estimates to more traditional methods. The differences in the correlations in this review for the different dietary assessment methods may be attributed to the differences in collection methods, such as FFQs, which provide better estimation of longer term intake, may reflect better dietary estimation of more habitual intakes than compared with single 24-h recalls. The majority of dietary assessments in the current review were food records which may be more sensitive to assessing details of dietary intake such as cooking methods and mode of consumption. 

Many of the studies were cross-sectional in design, meaning dietary intake and biomarkers were assessed at a single time point. Those studies which had a cohort design also only reported correlations at one time point. Whilst this was suited to the specific aim of studies examining associations between intake and biomarkers, depending on the dietary intake method, it is likely that the biomarker measurement and assessment of dietary intake may not reflect the same time period, i.e., use of an FFQ assessing intake over previous six months when most carotenoids have a half life of 1–2 months [55]. This may explain why many studies in the review used food records for improved compatibility with the biomarker assessment.

This review was limited to studies published in the English language and articles that were available via electronic databases. The review may be predisposed to a publication bias and an overrepresentation of studies that found positive associations between diet and plasma biomarkers. There were substantial levels of heterogeneity in the included studies. Major sources included variations in dietary assessment methods, the participant populations including sex, age and ethnicity, the range of plasma carotenoids assessed and the differing study protocols. Strengths to the review included the registered review methodology that adheres to the PRISMA guidelines for reporting of systematic reviews and the rigorous methodological process of obtaining the included studies that were extracted by two independent reviewers including quality checks to determine any bias and a standardized data extraction.

## 5. Conclusions

In conclusion, the current review provides an overview of technology-based dietary assessment methods that have been used in validation studies in comparison with plasma carotenoids as a biomarker of usual intake. Technology-based studies most commonly use retrospective measures of dietary assessment for comparison with carotenoid biomarkers. It was found that a wide variation in correlation values exists in the reviewed studies. The correlations were moderate and demonstrate that some of the technology-based dietary assessments can provide good estimates of carotenoid intake when compared to objective biomarkers of carotenoids. More validation studies that use technology-based dietary assessment methods with comprehensive nutrition reporting are required. 

## Figures and Tables

**Table 1:** Description of included studies.

Source	Country	Study Design	*n*	Gender	Age (Year)	Dietary Method + Reporting Period	Supplements Assessed	Dietary Carotenoids Assessed	Nutritional Database Used	Biochemical Carotenoids Assessed	Biochemical Method	Fasting Time Length
Arab et al. 2011 [25]	USA	Cohort	262	34.8% M	21–69	8 × 24-h recalls over two visits using web-based mutli pass method + 124 item diet history FFQ.	Yes	α-carotene, β-carotene, β-cryptoxanthin, lycopene, and the combined intakes of lutein and zeaxanthin.	USDA food composition database and National Cancer Institute database.	Lycopene, α-carotene, β-carotene, β-cryptoxanthin and combined lutein + zeaxanthin	HPLC	10 h fast
Bingham et al. 1995 [22,29,36,37]	UK	Cohort	160	100% F	50–65	4-day weighed food records at four timepoints over 12 months—two FFQs (each with 130 food items) were completed—27% of question related to vegetables in Cambridge FFQ and 18% in Oxford., two variants of the 24-h recall (structured/unstructured) and three types of food diary (7-day record + two checklists).	UC	β-carotene equivalents	Food tables	α-carotene, β-carotene, cis-carotnen, β-cryptoxanthin, lutein, lycopene,	Absorptiometric detection	Overnight fast
Dauchet et al. 2008 [32]	France	Cross sectional	3521	42% M	35–60	6 × 24-h dietary records	UC	F&V	NR	β-carotene	HPLC	Fasted
Faure et al. 2006 [33]	France	Cross sectional	12,741	39% M	F 35–60; M 45–60	6 × daily 24-h food records (4 week days and 2 weekend days)	UC	β-carotene	NR	β-carotene	HPLC	Fasted
Galan et al. 2005 [34]	France	Cross sectional	3128	42% M	F 35–60; M 45–60	6 × 24-h records over 18 months (4 week days and 2 weekend days	UC	β-carotene	French CIQUAL table + Mc Cance andWiddowson	β-carotene	HPLC	12 h
Kant et al. 2002 [35]	USA	Cross sectional	13,400	F 6948; M 6452	≥20	24-h recall	UC	NR specifically; F&V intake (in addition to various quantitative ax)	USDA	α-carotene, β-carotene, β-cryptoxanthin, lycopene, lutein/zeaxanthin	UC	Fasted
Kant et al. 2005 [26]	USA	Cross sectional	8719	49% M	≥20, <50 (5896); ≥50 (2764)	24-h recall + 3 diet quality scores: Healthy Eating Index (HEI); Recommended Food Score (RFS); Dietary Diversity Score (DDS-R)	UC	Diet Quality: HEI, RFS and DDS	NR	α-carotene, β-carotene, β-cryptoxanthin, lycopene, lutein/zeaxanthin	NR	Fasted
Lassale et al. 2016 [31]	France	Longitudinal cohort (3 weeks)	198	M 103 (52%) F 95 (48%)	Total 50.5 M 50.2 ± 16.2 F 50.7 ± 16.8	3 × dietary records	Yes	F&V	Nutrinet Sante composition table	β-carotene	β-carotene = HPLC	Fasting for at least 6 h
Van Lee et al. 2013 [30]	The Netherlands	Cross sectional	121	121	45–65	2 × non-consecutive 24-h recall, 180-item semi-quantitative FFQ.	Yes	F&V	Dutch Composition table	Alpha-carotene, β-cryptoxanthin, β-carotene, lutein, zeaxanthin	NR	Non-fasting
Pezdirc et al. 2015 [23]	Australia	Cross sectional	91	100% F	18.1–29.1	Australian Eating Survey 2010 (FFQ) 120 item reporting period 6 months	Yes	Alpha carotene, β-carotene, Lutein/zeaxanthin	Australian AusNut 1999 (all foods) revision 17 + AusFoods (brands) revision 5 (FoodWorks version 3.02.581)	Skin carotenoids: α-carotene, β-carotene, Lutein/zeaxanthin	CM700D specrophotometer)	Non fasting
Pierce et al. 2006 [24]	USA	Randomised trial	2922 (participants were from the WHEL study	100% F	18–70	Self-reported dietary intake using a set of four 24-h recalls over a 3 week period.	Yes	None, whole foods only. Food, juice and supplements	Minnesota Nutritional Data System software (Nutritional Data System version 4.01, 2001 University of Minnesota, Minneapolis, MN	α-carotene, β-carotene, β-cryptoxanthin, lutein + zeaxanthin, lycopene	HPLC	Fasting (unsure of time length)
Signorello et al. 2010 [27]	USA	Cross sectional	255 (125 AA, 130 non-Hispanic)	AA: F 63, M 62; Whites: F 64, M 66	40+	89-item FFQ. Nine items are specific to fruits or fruit juices, 13 are specific to vegetables.	Yes	α-carotene, β-carotene, β-cryptoxanthin, lutein+zeaxanthin, lycopene	nutrient databases developed for theSouthern Community Cohort study that were based on dietary patterns in the southern US.	α-carotene, β- carotene, β-cryptoxanthin, lutein + zeaxanthin, lycopene	HPLC	Non-fasted
Su et al. 2006 [28]	USA	Cross sectional	17,688	47% M	18–45 and 55+	24-h recall. Additional questions asked about use of vitamin and mineral supplements collected through verbal examination.	Yes	Salad, Vegetable	UC	α-carotene, β-carotene, lycopene	HPLC	UC

FFQ: food frequency questionnaire; UC: unclear; F&V: fruit and vegetable; NR: not reported; ax: assessment.

**Table 2:** Description of technology components of dietary measures from the included studies.

Reference	Technology-Based Dietary Assessment Method	Training for Participants	Device Used	Quantification of Portion Size	Record/Stand-Alone Software	Collection/Analysis
Arab et al. [25]	24-h recalls [38] to collect intake information using the multiple-pass method; Start of data collection/study 2006	The first three recalls were collected under supervision at assessment session, last three self-administered. Patients notified by email when recalls needed completion	NR	System contains 9349 foods. Images of foods (>7000) were displayed in a serving vessels and were used by participants to quantify amounts consumed.	Stand-alone software: Diet Day	Collection of previous day’s intake following the multiple-pass method in addition to programmed logic to skip irrelevant questions or branch to additional questions if required. Analysis automated using standard food and nutrient composition database (USDA). A reporting feature is also available comparing intake to national (US) nutrition recommendations
Bingham et al. [22,29]	Weighed food records; Start of data collection/study1985–1987	Subjects were visited in their homes the day before they were due to begin to weigh their food (day 0) they were given a demonstration of the PETRA scales and asked to try them out themselves. The following day (day 1), they were revisited and the verbal descriptions recorded on the tapes were checked for completeness using a personal cassette player. Subjects were left with written instructions and with a notebook for recording recipes and food eaten out of the home which had not been recorded on the PETRA scales.	Stand-alone device, Portable Electronic Tape Recorded Automatic (PETRA) scales. The PETRA console records verbal descriptions and weights of food (accurate to ±1 g) details of foods not disclosed to participant	Not required as WFR	Stand-alone software	Collection only: records then coded by hand for computerised calculation of nutrient intakes with food tables.
Dauchetet al. [32]	24-h Dietary Record; Start of data collection/study 1994	Participants were assisted by the conventional features of the software and an instruction manual was used for coding food portions	Completed on a small computerised terminal, the Minitel, provided to participants and commonly used in France.	Instructional manual with validated photograph >250 foods (>1000 generic foods) in seven portion sizes	Stand-alone ad hoc software available on the terminal	Collection and analysis using computerised food comp tables
Faure et al. [33]		Same as Dauchet et al.				
Galan et al. [34]		Same as Dauchet et al.				
Kant et al. [35]	24-h recall; Start of data collection/study 1988	Questionnaire completed at home with a medical exam which included diet in an interview in Mobile examination Centre (MEC)	micro-computer-based system	Recall aids, abstract food models, charts measuring cups and rulers to quantify foods consumed	Stand-alone software. Dietary Data Collection (DDC) system used structured probes within an open-ended interview question—See NHANES III [39]	DDC system facilitated standardised collection of dietary intake information automated coding and analysis.
Kant et al. [26]		Same as Kant et al. 2002				
Lassale et al. [31]	Dietary record; Start of data collection/study 2009	No training specified but study inclusion needed basic computer knowledge	Dedicated website with login and password to access on the day the web-based tool. The system is based on a secured interface designed by Medial expert system (MXS)	Picture booklet >250 generic foods (2000 individual foods) in seven different portion sizes		Collection + Analysis: The system has a food browser and then participants select a portion size using images taken from a previously validated picture book. The system includes prompting to assist in retrieving details of food. Nutrient intakes are calculated ad hoc using nutrient Sante composition tables that links the item in the survey to its nutrient content
Van Lee et al. [30]	24 Recall, multiple pass; Start of data collection/study: 2007 [40]	Interviewers were trained in interviewing techniques and using the EPIC software, a computerised 24-h recall that follows standardised procedures with a quick list and then provide details	Software installed on computer (Windows OS)	Portion sizes estimated using photos of household measures, standard units	Stand-alone software, EPIC-SOFT upgrade of original software [41]	Collection and Analysis: 24-h recalls were collected using EPIC soft Nutrient intakes calculated in software using Dutch food composition tables
Pezdirc et al. [23]	FFQ; Start of data collection/study: 2012	UC	UC web based	Standard portion sizes applied from national datasets		Collection only: FFQ data collected from online surveys not clear if nutrient analysis was computerised
Pierce et al. [24]	24 Recalls; Start of data collection/study: 1995	Participants were taught to estimate food portions and to describe specifics of foods	Stand-alone software; software driven protocol, 5 pass including quick list forgotten foods, time and occasion, details and final probes		Stand-alone software: Minnesota Nutritional Data System	Collection and Analysis: Multi-pass software driven protocol with nutrients estimated Unclear if automated—in particular back then. It appears that system is linked to a food comp database: http://www.ncc.umn.edu/products/
Signorelloet al. [27]	FFQ; Start of data collection/study 2002	UC	Computer-assisted	Does not directly assess portions, applies standard portions sizes developed for use in study		Collection and analysis: The 89 item FFQ was administered through a computer assisted in person interview conducted in a community health centre. Nutrients estimations were derived from sex and race specific databases developed for the study [42] Not clear if automated
Su et al. [28]	24R—see info for Kant above as also used NHANES III data	UC	UC web based	UC	Stand-alone Automated Dietary data Collection system	Collection and analysis: A interview included a 24-h recall system that was collected using the dietary data collection system. This was supplemented with interview questions about supplements and alcohol. Dietary data was aggregated and algorithms applied to reflect gram amounts

WFR: weighed food record.

**Table 3:** Outcomes of Included studies.

Source	Dietary Carotenoid Intake	Plasma Carotenoid Concentrations	Correlations between Diet and Plasma
Arab et al. [25]	Mean intake (ug/day) of carotenoids in African Americans (AA) and Whites (W) from 24HDR and DHQ. 24HDR: α-carotene (AA) 310, (W) 71; β-carotene (AA) 1420, (W) 2027; β-cryptoxanthin (AA) 110, (W) 120; Lutein + zeaxanthin (AA) 3420, (W) 4500; lycopene (AA) 3170, (W) 6320.	African Americans (Mean, μmol/L): α-carotene 0.06; β-carotene 0.28; β-cryptoxanthin 0.18; lutein + zeaxanthin 0.25; lycopene 0.60; Whites (Mean, μmol/L): α-carotene 0.07; β-carotene 0.31; β-cryptoxanthin 0.16; lutein + zeaxanthin 0.27; lycopene 0.57;	24HDR—Whites (AA): lutein+ zeaxanthin 0.48 (0.23), β-cryptoxanthin 0.51 (0.40), lycopene 0.13 (0.15), α-carotene 0.27 (0.18) β-carotene 0.38 (0.03); NCI–DHQ—Whites (AA): lutein+ zeaxanthin 0.47 (0.21), β-cryptoxanthin 0.33 (0.26), lycopene 0.02 (0.20), α-carotene 0.28 (0.24), β-carotene 0.31 (0.17)
Bingham et al. [22,29,36,37]	Five quintiles from PETRA-based WFR : Mean ± SE carotene g/day 1st (lowest) quintile 3.5 ± 0.3; 2nd 3.7 ± 0.4; 3rd 3.1 ± 0.3; 4th 3.7 ± 0.4; 5th (highest) 3.5±0.4; Total carotene: 1st–4th qunitile 3.5 ± 0.17.	Reported in Bingham 1995: Mean ± SE μmol/L: α-carotene 1st: 0.12 ± 0.02; 2nd: 0.13 ± 0.02; 3rd: 0.11 ± 0.01; 4th: 0.11 ± 0.02; 5th: 0.07 ± 0.01; β-carotene 1st: 0.57 ± 0.06; 2nd: 0.62 ± 0.07; 3rd: 0.50 ± 0.07; 4th: 0.49 ± 0.07; 5th 0.35 ± 0.04; cis-carotene 1st: 0.05±0.005; 2nd: 0.05±0.004; 3rd: 0.05±0.006; 4th: 0.04±0.005; 5th: 0.04±0.003; β-cryptoxanthin 1st: 0.26±0.02; 2nd: 0.28±0.04; 3rd: 0.29±0.03; 4th: 0.30±0.06; 5th: 0.24±0.04; lutein 1st: 0.45 ± 0.03; 2nd: 0.47 ± 0.04; 3rd: 0.39 ± 0.03; 4th: 0.44 ± 0.05; 5th 0.32 ± 0.03; lycopene 1st: 0.33 ± 0.02; 2nd: 0.36 ± 0.04; 3rd: 0.28 ± 0.03; 4th: 0.30 ± 0.06; 5th: 0.24 ± 0.04; Reported in Bingham 1997(26 suppl 1): Mean ± SD carotene (mg): 16-day weighed records 3.4 ± 1.9; FFQ 5.1 ± 3.2; 24-h recall 3.5 ± 3.7; 7-day estimated food record (food diary) 3.2 ± 1.8	1995 results: dietary β-carotene equivalents from PETRA WFR and plasma β-carotene (*r* = 0.48); α-carotene 0.62. lutein 0.36, cryptoxanthin 0.20, lycopene0.33; Comparison correlations between dietary intake and plasma (FFQ, 24-h recall, checklist) β-carotene 0.15, 0.08, 0.28 lutein 0.03, 0.05, 0.21, cryptoxanthin −0.02, −0.03, 0.07, Lycopene 0.17, 0.08, 0.21 α-carotene 0.42, 0.19, 0.34; 1997 results: correlations between dietary carotene and plasma β-carotene: weighed records *r* = 0.46; checklist *r* = 0.30; checklist with portions *r* = 0.27; oxford FFQ *r* = 0.15; cambridge FFQ *r* = 0.04; unstructured 24-h recall *r* = 0.09; structured 24-h recall *r* = 0.00.
Dauchet et al. [32]	Mean (SD) Vegetables + Fruits + Juices M: 416 (182) F: 465 (156) Vegetables M: 198 (87) F: 213 (80) Fruits + Fruit Juices: M: 218 (144) F: 242 (118) Fruits M: 180 (128) F: 199 (104) Fruit Juices: M: 37 (66) F: 43 (57)	μmol/L—Median (Range): Male β-carotene 0.33 (0.18–0.54); Female 0.44 (0.26–0.69)	Correlation values with β-carotene only range from 0.04 for fruit juices to 0.25 for Vegetables+ Fruit + juices
Faure et al. [33]	β-carotene (mg/day) Means ± SD MEN: 3140 ± 1540 (35–45 years); 4090 ± 2290 (45–50 years); 4110 ± 2310 (50–60 years); 4470 ± 2240 (60–63 years) WOMEN: 3790 ± 2152 (35–45 years); 3810 ± 2164 (45–50 years); 4120 ± 2216 (50–60 years); 4100 ± 1931 (60–63 years)	B carotene ug/day, Mean ± SD Females: 35–45 years 3790 ± 2152; 45–50 years 3810 ± 2164; 50-60 years 4120 ± 2216; 60–63 years 4100 ± 1931 Males: 35–45 years 3140 ± 1540; 45–50 years 4090 ± 2290; 50-60 years 4110 ± 2310; 60–63 years 4470 ± 2240	Regression analysis: estimated dietary intake and serum β-carotene b coefficient and SE 0.29 (0.02)
Galan et al. [34]	β-carotene (mg/day) Mean ± SD M 4.1 ± 2.5; F 4.0 ± 2.6	β-carotene (μmol/L) M 0.47 ± 0.35; F 0.67 ± 0.43	β-carotene *r* = 0.21, mean ± SE M 0.24 ± 0.03; F 0.22 ± 0.02
Kant et al. [35]	Amount of fruit (g): first tertile of energy intake, Healthy weight M 141 ± 12; F 120 ± 12; third tertile M 236 ± 17; F 193 ± 9. Number of foods from fruit: first tertile of energy intake, Healthy weight M 0.8 ± 0.04; F 1.0 ± 0.1 third tertile M 1.04 ± 0.1; F 1.04 ± 0.1 Amount of vegetables (g) first tertile of energy intake, Healthy weight	nmol/L reported by tertiles of energy intake by BMI catergory (HW reported here) Serum beta-carotene: M: 0.36 ± 0.02, F: 0.51 ± 0.03 Serum alpha-carotene: M: 0.09 ± 0.008, F: 0.12 ± 0.006 Serum beta-cryptoxanthin: M: 0.15 ± 0.006, F: 0.18 ± 0.007 Serum lutein-zeaxanthin: M: 0.37 ± 0.01, F: 0.41 ± 0.013 Serum lycopene: M: 0.41 ± 0.016, F: 0.40 ± 0.013	Dietary carotenoids were positive predictors of α-, β-carotene, β-cryptoxanthin and lutein zeaxanthin (*p* < 0.001)
Kant et al. [26]	The mean HEI 63.75, RFS 3.97 and DDS-R 2.44	*n* = 7997. Dietary scores (HEI, RFS, DSS-R) split into quartiles and mean ± SEM reported for each (µmol/L) serum α-carotene C1: 0.072 ± 0.002,0.072 ± 0.003, 0.073 ± 0.002; C2: 0.083 ± 0.002, 0.077 ± 0.002, 0.085 ± 0.002 C3: 0.093 ± 0.003, 0.084 ± 0.002, 0.092 ± 0.002; C4: 0.118 ± 0.004, 0.114 ± 0.003, 0.119 ± 0.004 β ± SE^2^: 0.001 ± 0.000, 0.009 ± 0.000, 0.012 ± 0.001 β-carotene C1: 0.322 ± 0.009, 0.326 ± 0.010, 0.330 ± 0.008; C2: 0.359 ± 0.013, 0.340 ± 0.009, 0.357 ± 0.008 C3: 0.373 ± 0.010, 0.354 ± 0.010, 0.374 ± 0.011; C4: 0.441 ± 0.014, 0.438 ± 0.008, 0.454 ± 0.013 β ± SE^2^: 0.003 ± 0.000, 0.022 ± 0.002, 0.035 ± 0.004 β-cryptoxanthin C1: 0.143 ± 0.003, 0.139 ± 0.004, 0.142 ± 0.003; C2: 0.158 ± 0.004, 0.148 ± 0.004, 0.158 ± 0.004 C3: 0.165 ± 0.003, 0.162 ± 0.004, 0.172 ± 0.004; C4: 0.196 ± 0.005, 0.190 ± 0.004, 0.193 ± 0.006 β ± SE^2^: 0.001 ± 0.000, 0.009 ± 0.000, 0.014 ± 0.001 Lutein/zeaxanthin C1: 0.351 ± 0.006, 0.335 ± 0.005, 0.345 ± 0.003; C2: 0.367 ± 0.007, 0.343 ± 0.009, 0.372 ± 0.006 C3: 0.386 ± 0.008, 0.370 ± 0.006, 0.389 ± 0.008; C4: 0.411 ± 0.008, 0.424 ± 0.008, 0.413 ± 0.011 β ± SE^2^: 0.002 ± 0.000, 0.016 ± 0.001, 0.019 ± 0.004 Lycopene C1: 0.440 ± 0.005, 0.443 ± 0.007, 0.443 ± 0.006; C2: 0.456 ± 0.006, 0.433 ± 0.007, 0.439 ± 0.007 C3: 0.443 ± 0.007, 0.446 ± 0.007, 0.442 ± 0.006; C4: 0.449 ± 0.007, 0.456 ± 0.006, 0.466 ± 0.007 β ± SE^2^: 0.000 ± 0.000, 0.002 ± 0.001, 0.006 ± 0.002	All three dietary scores were strong positive predictors of all serum carotenoids, excpet lycopene (sig for RFS and DDS-R only *p* < 0.05). Pearson’s *r* with Carotene (RE): HEI = 0.20; RFS = 0.31; DDS-R = 0.19; all *p* < 0.0001
Lassale et al. [31]	Mean (95% CI) Male: Fruit g/day: 207.6 (178.3–236.8); Vegetables: 244.9 (220.9–268.9); β-carotene µg/day: 4175.6 (3594.5–4756.8) Female: Fruit: 185.8 (155.4–216.2); Vegetables: 228.8 (203.8–253.8); β-carotene: 3562.5 (2957.3–4167.6)	Beta-carotene (ug/dL) Male: Geometric unadjusted mean (95% CI): 40.01 (34.42–45.59); Adjusted mean (95% CI): 40.92 (34.05–47.79) Female (*n* = 95): Geometric unadjusted mean (95% CI): 45.16 (39.31–50.96); Adjusted mean (95% CI): 46.02 (39.52–52.46)	*r* (95% CI) Crude Correlations M: F&V and β-carotene: 0.46 (0.29, 0.60); Fruit and β-carotene: 0.35 (0.17, 0.51); Veg and β-carotene: 0.38 (0.21, 0.54) F: F&V and β-carotene: 0.37 (0.19, 0.54); Fruit and B-carotene: 0.41 (0.22, 0.56); Veg and β-carotene: 0.24 (0.04, 0.42) Adjusted Correlations M: F&V and β-carotene: 0.35 (0.16, 0.52); Fruit and β-carotene: 0.29 (0.10, 0.47); Veg and β-carotene: 0.29 (0.10, 0.47) F: F&V and β-carotene: 0.41 (0.22, 0.57); Fruit and β-carotene: 0.36 (0.17, 0.53); Veg and β-carotene: 0.37 (0.17, 0.53) nutrient reported intake and corresponding plasma biomarkers; Crude correlations β-carotene M 0.47 (0.31, 0.61); F 0.37 (0.18, 0.53) Adjusted correlations β-carotene M 0.38 (0.20, 0.54); F 0.37 (0.17, 0.53)
Van Lee et al. [30]	Median (IQR), 24-h recall: Vegetables: 8.8 (3.3); Fruit: 10.0 (3.9) FFQ: Vegetables: 6.3 (5.2); Fruit: 10.0 (4.4)	Organised into tertiles: T1 *n* = 40; T2 *n* = 41; T3 *n* = 40. Mean (SD) for carotenoids (μg/100mL): T1 = 114.4 (89.1); T2 = 113.8 (84.7); T3 = 128.7 (90.2)	Correlation/*r* (95%CI) for serum carotenoids and Vegetables (24-h recall): 0.25 (0.07–0.41); Vegetables (FFQ): 0.17 (−0.01, 0.34); Fruit (24 hr recall): 0.09 (−0.09, 0.27); Fruit (FFQ): 0.25 (0.08, 0.41)
Pezdirc et al. [23]	Median (IQR) in μg/day: α-carotene: 1988.6 (1220.2–2611.6); β-carotene: 6872.4 (4462.6–8918.6); Lutein zeaxanthin: 2276.8 (1523.6–2895.1); Lycopene: 5054.8 (2975.1–7488.5); Median (IQR) in servings/day: Total fruit intake: 1.8 (1.0–2.7); Total vegetable intake: 3.8 (2.7–5.2); Total F&V intake: 5.9 (4.1–7.4)	Skin carotenoids: L* 65.2 ± 2.1, a* (redness) 9.3 ± 1.2, b* (yellowness) 16.3 ± 2.1	B coefficient ± SE Skin a*: Fruit 0.8 ± 0.3, vegetables 0.6 ± 0.3, F+V 0.7 ± 0.2; b* fruit 1.8 ± 0.4, vegetables 1.4 ± 0.4, F+V 1.5 ± 0.4. Relationship between veg intake and skin reflectance (wavelengths 400–540 nm) negatively correlated with absorption spectra of lycopene. F&V intake and skin reflectance—vely correlated with absorption spectra of β-carotene, lycopene, and mean carotenoid.
Pierce et al. [24]	Dietary intakes reported as relative contributions of food juice and supplements to plasma carotenoids, not absolute amounts	*Log transformed* (μmol/L) Mean (SD): Intervention group: Baseline, [12mo]: α-carotene 0.204 (0.230), [0.597 (0.686)]; β-carotene 0.865 (0.874), [1.466 (1.416)]; β-cryptoxanthin 0.171 (0.155), [0.179 (0.159)]; lutein+zeaxanthin 0.380 (0.200), [0.459 (0.243)]; lycopene 0.653 (0.345), [0.739 (0.368)]; Total 2.272 (1.294), [3.440 (2.320)]. Comparison group: Baseline, [12mo]: α -carotene 0.204 (0.213), [0.203 (0.219)]; β-carotene 0.914 (1.065), [0.868 (0.937)]; β-cryptoxanthin 0.178 (0.175), [0.177 (0.157)] lutein+zeaxanthin 0.376 (0.204), [0.381 (0.213)]; lycopene 0.655 (0.344), [0.650 (0.340)]; Total 2.327 (1.470), [2.279 (1.371)]	Full model β coefficients: Juice: α-carotene 0.083 (*p* < 0.001), β-carotene 0.011 (*p* < 0.001), lutein+zeaxanthin 0.005 (*p* < 0.05), lycopene 0.018 (*p* < 0.001). Food: α-carotene 0.074 (*p* < 0.001), β-carotene 0.135 (*p* <0.001), lutein + zeaxanthin 0.096 (*p* < 0.001), lycopene 0.034 (*p* < 0.001). Supplement: β-carotene 0.040 (*p* < 0.001), lutein + zeaxanthin 0.017 (*p* < 0.001)
Signorello et al. [27]	*All log transformed* (μg/day) Mean* (SD) where * is *p* < 0.05 for 2-sample *t*-test comparing mean values by race within each sex. AA female: α-carotene 666.0* (787.5), β- carotene 5820.8* (5119.8), β-cryptoxanthin 264.1* (213.5), lutein+zeaxanthin 5025.2* (4934.4), lycopene 4892.1 (5301.7). AA male: α-carotene 556.2 (533.1), β- carotene 6212.2* (5750.7), β-cryptoxanthin 298.6* (263.4), lutein + zeaxanthin 5497.4* (5769.8), lycopene 6994.7 (5098.4). White female: α-carotene 419.0* (364.3), β-carotene 3203.4* (1952.5), β-cryptoxanthin 160.5* (164.0), lutein + zeaxanthin 2223.4* (1375.1), lycopene 4050.9 (2979.3). White male: α-carotene 572.2 (483.9), β-carotene 3617.0* (2656.1), β-cryptoxanthin 175.7* (150.6), lutein + zeaxanthin 2583.7* (2199.7), lycopene 6949.9 (5299.3)	*All log transformed, except lycopene which is square root transformed* (μg/dL) Mean* (SD) where * is *p* < 0.05 for 2-sample *t*-test comparing mean values by race within each sex. AA F: α-carotene 4.4* (4.8), β-carotene 21.3* (20.6), β-cryptoxanthin 10.7* (6.7), lutein+zeaxanthin 21.8* (10.4), lycopene 28.5 (12.7). AA M: α-carotene 2.7 (2.6), β-carotene 13.1 (11.1), β-cryptoxanthin 8.2 (5.6), lutein+zeaxanthin 20.9* (10.0), lycopene 33.4 (17.2). White F: α-carotene 2.7* (2.0), β-carotene 13.8* (12.6), β-cryptoxanthin 6.4* (4.3), lutein+zeaxanthin 14.3* (6.3), lycopene 31.1 (13.6). White M: α-carotene 3.7 (4.8), β-carotene 11.2 (9.9), β-cryptoxanthin 6.9 (4.1), lutein+zeaxanthin 15.3* (7.0), lycopene 33.8 (14.8)	α-carotene 0.32 (*p* < 0.001), β-carotene 0.25 (*p* < 0.001), β-cryptoxanthin 0.37 (*p* < 0.001), lutein + zeaxanthin 0.35 (*p* < 0.001), lycopene 0.18 (P < 0.01)
Su et al. [28]	Salad consumption Mean ± SD (g/day): 18–45 years: F 39.2 ± 82.3; M 40.0 ± 90.1; 55+ years: F 36.1 ± 76.6; M 37.7 ± 83.1; Vegetable consumption Mean ± SD (g/day) 18–45 years: Females 33.6 ± 75.2; Males 36.0 ± 82.3; 55+ years: Females 31.3 ± 71.8; Males 32.7 ± 77.6	Mean serum levels by level (L = low, M = medium, H = high) of salad/vegetable consumption (μg/dL): α-carotene salad: F (L) 4.84; (M) 4.84; (H) 5.91; M (L) 3.76; (M) 4.30; (H) 4.84; α-carotene vegetables: F (L) 4.84; (M) 4.84; (H) 5.81; M (L) 3.76; (M) 4.30; (H) 4.84; β-carotene salad: F (L) 20.97; (M) 20.97; (H) 24.73; M (L) 16.13; (M) 18.28; (H) 19.35; β-carotene vegetables:F (L) 20.97; (M) 20.97; (H) 24.73; M (L) 16.13; (M) 18.28; (H) 19.35; lycopene salad: F (L) 20.43; (M) 21.51; (H) 23.12; M (L) 21.51; (M) 23.13; (H) 24.19; lycopene vegetables: F (L) 20.43; (M) 21.51; (H) 23.12; M (L) 21.51; (M) 23.13; (H) 24.19	There was a positive relationship between consumption (salad and vegetable) and serum carotenoids for females and males: Salad: α-carotene F 1.24; M 1.35; β-carotene F 1.06; M 1.27; lycopene F 1.19; M 1.15; Vegetables: α-carotene F 1.26; M 1.31; β-carotene F 1.21; M 1.26; lycopene F 1.18; M 1.12

24HDR: 24-h diet recall; DHQ: diet history questionnaire; NCI: national cancer institute.

## Supplementary Materials

The following are available online at http://www.mdpi.com/2072-6643/9/2/140/s1, Table S1: Study Quality.

## References

[1] Rollo M.E., Williams R.L., Burrows T., Kirpatrick S.I., Bucher T., Collins C.E. (2016). What Are They Really Eating? A Review on New Approaches to Dietary Intake Assessment and Validation. Curr. Nutr. Rep..

[2] Gemming L., Utter J., Mhurchu C.N. (2015). Image-assisted dietary assessment: A systematic review of the evidence. J. Acad. Nutr. Diet..

[3] Illner A.K., Freisling H., Boeing H., Huybrechts I., Crispim S., Slimani N. (2012). Review and evaluation of innovative technologies for measuring diet in nutritional epidemiology. Int. J. Epidimiol..

[4] Norat T., Aune D., Chan D., Romaguera D. (2014). Fruits and Vegetables: Updating the Epidemiologic Evidence for the WCRF/AICR Lifestyle Recommendations for Cancer Prevention. Cancer Treat. Res..

[5] Romaguera D., Vergnaud A., Peeters P., van Gils C., Chan D., Ferrari P., Clavel-Chapelon F.F.G., Perquier F., Kaaks R., Teucher B. (2012). Is concordance with World Cancer Research Fund/American Institute for Cancer Research guidelines for cancer prevention related to subsequent risk of cancer? Results from the EPIC study. Am. J. Clin. Nutr..

[6] Etminan M., Takkouche B., Caamano-Isorna F. (2004). The role of tomato products and lycopene in the prevention of prostate cancer: A meta-analysis of observational studies. Cancer Epidemiol. Biomark. Prev..

[7] Koushik A., Hunter D.J., Spiegelman D., Beeson W.L., van den Brandt P.A., Buring J.E., Calle E.E., Cho E., Fraser G.E., Freudenheim J.L. (2007). Fruits, vegetables, and colon cancer risk in a pooled analysis of 14 cohort studies. J. Natl. Cancer Inst..

[8] Lam T.K., Gallicchio L., Lindsley K., Shiels M., Hammond E., Tao X.G., Chen L., Robinson K.A., Caulfield L.E., Herman J.G., Guallar E. (2009). Cruciferous vegetable consumption and lung cancer risk: A systematic review. Cancer Epidemiol. Biomark. Prev..

[9] Dauchet L., Amouyel P., Hercberg S., Dallongeville J. (2006). Fruit and vegetable consumption and risk of coronary heart disease: A meta-analysis of cohort studies. J. Nutr..

[10] He F.J., Nowson C.A., Lucas M., MacGregor G.A. (2007). Increased consumption of fruit and vegetables is related to a reduced risk of coronary heart disease: Meta-analysis of cohort studies. J. Hum. Hypertens..

[11] Dauchet L., Amouyel P., Dallongeville J. (2005). Fruit and vegetable consumption and risk of stroke: A meta-analysis of cohort studies. Neurology.

[12] He F.J., Nowson C.A., MacGregor G.A. (2006). Fruit and vegetable consumption and stroke: Meta-analysis of cohort studies. Lancet.

[13] Villegas R., Shu X.O., Gao Y.T., Yang G., Elasy T., Li H., Zheng W. (2008). Vegetable but not fruit consumption reduces the risk of type 2 diabetes in Chinese women. J. Nutr..

[14] Hamer M., Chida Y. (2007). Intake of fruit, vegetables, and antioxidants and risk of type 2 diabetes: Systematic review and meta-analysis. J. Hypertens..

[15] Seyedrezazadeh E., Moghaddam M., Ansarin K., Vafa M.R., Sharma S., Kolahdooz F. (2014). Fruit and vegetable intake and risk of wheezing and asthma: A systematic review and meta-analysis. Nutr. Rev..

[16] Wood L., Garg M., Smart J., Scott H., Barker D., Gibson P. (2012). Manipulating antioxidant intake in asthma: A randomized controlled trial. Am. J. Clin. Nutr..

[17] Peto R., Doll R., Buckley J., Sporn M. (1981). Can dietary beta carotene materially reduce human cancer rates?. Nature.

[18] Burrows T., Williams R., Rollo M., Wood L., Garg M., Jensen M., Collins C. (2015). Plasma carotenoid levels as biomarkers of dietary carotenoid consumption: A systematic review of the validation studies. J. Nutr. Intermed. Metab..

[19] Burrows T.L., Hutchesson M.J., Rollo M.E., Boggess M.M., Guest M., Collins C.E. (2015). Fruit and vegetable intake assessed by food frequency questionnaire and plasma carotenoids: A validation study in adults. Nutrients.

[20] Burrows T.L., Warren J.M., Colyvas K., Garg M.L., Collins C.E. (2009). Validation of overweight children’s fruit and vegetable intake using plasma carotenoids. Obesity.

[21] Association A.D. (2008). Evidence Analysis Manual: Steps in the ADA Evidence Analysis Process.

[22] Bingham S.A., Cassidy A., Cole T., Welch A., Runswick S., Black A., Thurnham D., Bates C., Khaw K., Key T. (1995). Validation of weighed recrods and other methods of dietary assessment using the 24 h urine nitrogen techniqie and other biological markers. Br. J. Nutr..

[23] Pezdirc K., Hutchesson M.J., Whitehead R., Ozakinci G., Perrett D., Collins C.E. (2015). Fruit, Vegetable and Dietary Carotenoid Intakes Explain Variation in Skin-Color in Young Caucasian Women: A Cross-Sectional Study. Nutrients.

[24] Pierce J., Natarajan L., Sun S. (2006). Increases in plasma carotenoid concentrations in response to am ajor dietary change in the womens healthy eating and living study. Cancer Epidemiol. Biomark. Prev..

[25] Arab L., Cambou M., Craft N., Wesseling-Perry K., Jardack P., Ang A. (2011). Racial differences in correlations between reported dietary intakes of carotenoids and their concentration biomarkers. Am. J. Clin. Nutr..

[26] Kant A., Graubard B. (2005). A comparison of three dietary pattern indexes fro predicting biomarkers of diet and disease. J. Am. Coll. Nutr..

[27] Signorello L., Buchowski M., Cai Q., Munro H., Hargreaves M., Blot W. (2010). Biochemical validation of a food frequency questionniare estimated carotenoid a tocopherol and folate intakes among african americans and non hispanic whites in the Southern Community cohort study. Am. J. Epidemiol..

[28] Su L., Arab L. (2006). Salad and Raw vegetable consumption and nutrtional status in the adult US population: Results from the third National Health and Nutrition Examination Survey. J. Am. Diet. Assoc..

[29] Bingham S.A., Day N. (1997). Using biochemical markers to assess validity of prosepctive dietary assessment methods and the effect of energy adjustment. Am. J. Clin. Nutr..

[30] Van Lee L., Feskens E.J., van Huysduynen E.J.C.H., de Vries J.H.M., van’t Veer P., Geelen A. (2013). The Dutch Healthy Diet index as assessed by 24 h recalls and FFQ: Associations with biomarkers from a cross-sectional study. J. Nutr. Sci..

[31] Lassale C., Castetbon K., Laporte F., Deschamps V., Vernay M., Camilleri G.M., Faure P., Hercberg S., Galan P., Kesse-Guyot E. (2016). Correlations between Fruit, Vegetables, Fish, Vitamins, and Fatty Acids Estimated by Web-Based Nonconsecutive Dietary Records and Respective Biomarkers of Nutritional Status. J. Acad. Nutr. Diet..

[32] Dauchet L., Peneau S., Bertrais S., Vergnaud A.C., Estaquio C., Kesse-Guyot E., Czernichow S., Favier A., Faure H., Galan P. (2008). Realtionships between different types of fruit and vegetable consumption and serum concentrations of antioxidant vitamins. Br. J. Nutr..

[33] Faure H., Preziosi P., Rousell A., Bertrais S., Galan P., Hercberg S., Favier A. (2006). Factors influencing blood concentration of retinol, a-tocopherol, vitamin C and B carotene in french participants in the SU.VI.max trial. Eur. J. Clin. Nutr..

[34] Galan P., Viteri F.E., Bertrais S., Czernichow S., Faure H., Arnuad J., Ruffieux D., Chenal S., Arnualt N., Favier A. (2005). Serum Concentrations of B carotene, vitamins C and E, Zinc, selenium, are influenced by sex, age, diet and smoking status, alcohol consumption and corpulence in a general French adult population. Eur. J. Clin. Nutr..

[35] Kant A. (2002). Nature of dietary reporting by adults in the third NAtional Health and Nutrition Examination survey, 1988–1994. J. Am. Coll. Nutr..

[36] Bingham S.A. (1997). Dietary assessments in the European prospective study of diet and cancer (EPIC). Eur. J. Cancer Prev..

[37] Bingham S.A., Day N. (1997). Validation of dietary assessment methods in the UK arm of EPIC using weighed records, and 24-h urinary nitrogen and potassium and serum vitamin C and carotenoids as biomarkers. Int. J. Epidemiol..

[38] Arab L., Wesseling-Perry K., Jardack P., Henry J., Winter A. (2010). Eight Self-Administered 24-h Dietary Recalls Using the Internet are Feasible in African Americans and Caucasians: The Energetics Study. J. Am. Diet. Assoc..

[39] National Center for Heaith Statistics Plan and operation of the Third National Heaith and Nutrition Examination Survey, 1988–94. https://www.cdc.gov/nchs/data/series/sr_01/sr01_032.pdf.

[40] Crispim S.P., Geelen A., de Vries J.H.M., Freisling H., Souverein O.W., Hulshof P.J.M., Ocke M.C., Boshuizen H., Andersen L.F., Ruprich J. (2012). Bias in protein and potassium intake collected with 24-h recalls (EPIC-Soft) is rather comparable across European populations. Eur. J. Nutr..

[41] Slimani N., Casagrande C., Nicolas G., Freisling H., Huybrechts I., Ocké M.C., Niekerk E.M., van Rossum C., Bellemans M., de Maeyer M. (2011). The standardized computerized 24-h dietary recall method EPIC-Soft adapted for pan-European dietary monitoring. Eur. J. Clin. Nutr..

[42] Signorello L., Munro H., Buchowski M., Schlundt D., Cohen S., Hargreaves M., Blot W. (2009). Estimating Nutrient Intake From a Food Frequency Questionnaire: Incorporating the Elements of Race and Geographic Region. Am. J. Epidimiol..

[43] ABS 8146.0—Household Use of Information Technology, Australia, 2014–2015. http://www.abs.gov.au/ausstats/abs@.nsf/mf/8146.0.

[44] Stumbo P.J. (2013). New technology in dietary assessment: A review of digital methods in improving food record accuracy. Proc. Nutr. Soc..

[45] Bingham S.A. (1987). The dietary assessment of individuals: Methods, accuracy, new techniques and recommendations. Nutr. Abstr. Rev..

[46] Thompson F., Subar A., Loria C., Reedy J., Baranowski T. (2011). Need for Technological Innovation in Dietary Assessment. J. Acad. Nutr. Diet..

[47] Burrows T., Martin R., Collins C. (2010). A systematic review of the validity of dietary assessment methods in children when compared with the method of doubly labelled water. J. Am. Diet. Assoc..

[48] National Cancer Institute Automated Self-Administered 24-h (ASA24^®^) Dietary Assessment Tool. http://epi.grants.cancer.gov/asa24/.

[49] NCI National Cancer Institute. https://epi.grants.cancer.gov/asa24/.

[50] Innovation U. (2015). How Mobile Phones Are Changing the Developing World. https://blogs.unicef.org/innovation/how-mobile-phones-are-changing-the-developing-world/.

[51] Burrows T.L., Khambalia A., Perry R., Carty D., Hendrie G., Allman-Farinelli M., Garnett S., McNaughton S., Rangan A.M., Truby H. (2015). Great ‘app-eal’but not there yet: A review of iPhone nutrition applications relevant to child weight management. Nutr. Diet..

[52] Lunden I. 6.1B Smartphone Users Globally By 2020, Overtaking Basic Fixed Phone Subscriptions. http://techcrunch.com/2015/06/02/6-1b-smartphone-users-globally-by-2020-overtaking-basic-fixed-phone-subscriptions/.

[53] NCI Dietary Assessment Primer. https://dietassessmentprimer.cancer.gov/.

[54] Lachat C., Hawwash D., Ocké M.C., Berg C., Forsum E., Hörnell A., Larsson C., Sonestedt E., Wirfält E., Åkesson A. (2016). Strengthening the Reporting of Observational Studies in Epidemiology Nutritional Epidemiology (STROBE-nut): An Extension of the STROBE Statement. PLoS Med..

[55] Burri B., Neidlinger T., Clifford A. (2001). Serum carotenoid depletion follows first-order kinetics in healthy adult women fed naturally low carotenoid diets. J. Nutr..

