# Supplementary material for: A Systematic Review of Technology-Based Dietary Intake Assessment Validation Studies That Include Carotenoid Biomarkers
Source: Nutrients. 2017 Feb 14;9(2):140. doi: 10.3390/nu9020140 (PMC5331571; doi:10.3390/nu9020140)
Supplement: Supplementary file 1 [file nutrients-09-00140-s001.docx]

**Supplementary Materials: A Systematic Review of Technology-Based Dietary Intake Assessment Validation Studies That Include
Carotenoid Biomarkers**

Tracy L. Burrows, Megan E. Rollo, Rebecca Williams, Lisa G. Wood, Manohar L. Garg, Megan Jensen and Clare E. Collins

**Table S1.** Study Quality (Study Quality assessed using the American Dietetic Association tool).

| **Study (1st Author, Year)** | **Questions *** | | | | | | | | | | **Overall Quality** |
| --- | --- | --- | --- | --- | --- | --- | --- | --- | --- | --- | --- |
|  | **1** | **2** | **3** | **4** | **5** | **6** | **7** | **8** | **9** | **10** |  |
| Arab et al. (2011) | Y | Y | N | Y | NA | NA | Y | Y | Y | Y | N |
| Bingham et al. (1995, 1997) | Y | N | NA | N | NA | NA | Y | Y | Y | Y | N |
| Dauchet et al. (2008) | Y | Y | NA | Y | NA | NA | Y | Y | Y | Y | P |
| Faure et al. (2006) | Y | Y | NA | NA | NA | NA | Y | Y | Y | UC | P |
| Galan et al. (2005) | Y | Y | N | Y | UC | Y | Y | Y | Y | Y | P |
| Kant et al. (2002) | Y | Y | N/A | N/A | N/A | N | Y | Y | Y | Y | N |
| Kant et al. (2005) | Y | Y | N/A | N/A | N/A | N | Y | Y | Y | Y | N |
| Lassale et al. (2016) | Y | Y | N/A | N/A | N/A | Y | Y | Y | Y | Y | P |
| Van Lee et al. (2013) | Y | Y | N/A | N/A | N/A | N/A | Y | Y | Y | Y | P |
| Pezdirc et al. (2015) | Y | Y | N/A | Y | N/A | N/A | Y | Y | Y | Y | P |
| Pierce et al. (2006) | Y | N | Y | N | N | N | Y | Y | N | Y | N |
| Signorello et al. (2010) | Y | Y | Y | NA | N | Y | Y | Y | N | Y | P |
| Su et al. (2006) | Y | Y | NA | NA | NA | NA | Y | Y | N | N | N |

* The questions are as below: 1: *Was the research question clearly stated?* 2*: How was the sample selected (selection bias)?* 3: *Were study groups comparable?* 4*: Was the method of handling withdrawals described?* 5: *Was blinding used to prevent introduction of bias?* 6: *Were intervention/therapeutic regimens/exposure factor or procedure and any comparison(s) described in detail?* 7: *Were outcomes clearly defined and the measurements valid and reliable?* 8: *Was the statistical analysis appropriate?* 9: *Were conclusions supported by results with biases and limitations?* 10: *Is bias due to the study’s funding or sponsorship unlikely?*P = positive, N = neutral.
